# A Balancing Act During Covid-19: Teachers' Self-Efficacy, Perception of Stress in the Distance Learning Experience

**DOI:** 10.3389/fpsyg.2021.644108

**Published:** 2021-05-06

**Authors:** Emanuela Rabaglietti, Lynda S. Lattke, Beatrice Tesauri, Michele Settanni, Aurelia De Lorenzo

**Affiliations:** Department of Psychology, University of Turin, Turin, Italy

**Keywords:** distance learning, perceived stress, self-efficacy, transversal/soft skills, teacher-student relationship, COVID-19

## Abstract

One of the many drastic changes caused by Covid-19 was the quick implementation of distance learning which represented a great technological challenge to many teachers and students. In fact, Italy ranks 24th amongst the 27-EU member countries in digital competitiveness which testifies to the significant delays and gaps in basic digital skills amongst the population. Based on the difficulties encountered in organizing distance learning, we assumed that teachers' perceived stress increased. Given that transversal skills can be associated with this relationship, we hypothesized that among these skills, self-efficacy mediated the relationship between the difficulties in organizing distance learning and perceived stress. Since we targeted teachers from Italy and other European countries, we also hypothesized that this mediator effect would be different for both samples. Our sample was composed of a total of 366 primary/middle school teachers of which 86% female. After doing a mediation analyses with Process, Hayes' Model 4, we confirmed H1 but not H2: resulting in a partial mediation effect of self-efficacy for each individual group of teachers. Since difficulties of distance learning can affect the perception of stress, we believe that the promotion of transversal skills, such as self-efficacy, can better equip teachers when facing stressful situations.

## Introduction

Northern Italy was one of the first European areas to be severely affected by Covid-19 in a very short time (Armocida et al., 2020; JRC Map., 2020; Spiteri et al., 2020). In order to contain the spread of the virus, and as a way to ensure physical distancing, the Italian government issued a total lockdown becoming the first European government to take such measurement (Flaxman et al., 2020). This meant people were not allowed to leave their homes unless it was strictly necessary. As the virus started to spread, many other countries enacted restrictive containment measurements (European Union., 2020; JRC Map., 2020). From all closures, schools have taken a great toll for they had to ensure some form of distance learning (Buccolo et al., 2020; Kim and Asbury, 2020; Remuzzi and Remuzzi, 2020; UNESCO, 2020). Teachers and students were forced to quickly shift to a completely new method to continue their school program by relying on any means they had available at the time (Cowden et al., 2020; Daniel, 2020; Telli, 2020). This experience was shaped by many factors: from level of technological knowledge/resources (König et al., 2020) to drastic changes in lifestyle further enhanced by a sense of uncertainty (Hawryluck et al., 2004; Berinato, 2020; Brooks et al., 2020). All these situations generated stress which varied depending on teachers' perception and how well-equipped they were with transversal/soft skills (Montgomery and Rupp, 2005; Betoret, 2006). These skills such as self-regulation and self-efficacy are known for helping “*understand and manage emotions, set goals* amongst others” (Elias et al., 1997). Teachers who have these skills are able to cope better with new and unexpected situations of high stress (Carson and Runco, 1999; Betoret, 2006; Schwarzer and Hallum, 2008; Greenberg et al., 2016).

During Covid-19, changing schools from a physical place to a virtual space, highlighted some of these complexities. The school community was not prepared for this new way of teaching. In fact, while working and studying from home (Dhawan, 2020; Palareti, 2020; Reimers and Schleicher, 2020), teachers and students suddenly found themselves having to learn very quickly how to use digital tools they were barely familiar with, often encountering various types of problems as well as difficulty in guaranteeing access and the necessary resources (European Commission, 2020; Palareti, 2020). This method also meant having an appropriate space to work/study (Cowden et al., 2020; Filandri and Semi, 2020) and a number of devices available to family members who may need them at the same time (Lucisano, 2020; Ranieri et al., 2020; UNESCO, 2020). These difficulties in organizing distance learning became a source of stress for many teachers (Cowden et al., 2020; Palareti, 2020; Ziebell et al., 2020).

Before the pandemic, teaching was already known as an occupation with various sources of difficulties and of stress (Harmsen et al., 2018). This stress spectrum is wide: from classroom management (Klassen and Chiu, 2010) to policy changes which affect “*teaching methods, the content of the school curriculum and Assessment procedures*” (Kyriacou, 2001, p. 32) to workload (Travers and Cooper in Johnson et al., 2005; Jepson and Forrest, 2006; Ravichandran and Rajendran, 2007) which can affect family dynamics especially when there is pressure to re-distribute work and family time (Muirhead, 2000; Duxbury and Higgins, 2012) and furthermore if there are family problems (Palmer et al., 2012).

In this health pandemic period, many of these difficulties seemed to have come together all at once, along with the stress caused by teachers' need to suddenly change their method and move toward online teaching while lacking the necessary training (König et al., 2020). Muirhead (2000) has reported on the stress generated in teachers who were expected to create online courses and had not had any form of pre-service training on distance learning: online teaching in itself was also perceived as additional work for it meant possibly working at any moment of day or/and night including weekends, and “…*providing technological support*” (p.320) to students and families who were less familiar with these tools. During Covid-19, this scenario was further confirmed (Cowden et al., 2020) with the addition of an increase in screen time, which has generated even further stress (Ziebell et al., 2020) known as “*technostress*” and which has affected teaching performance (Christian et al., 2020). Yet independent of the type of stress, stress itself may be perceived differently by each person. This can depend on a number of factors such as gender (Mazure, 1998), socio-cultural and economic context (Vallejo et al., 2018) and transversal/soft skills, such as self-efficacy. Lee et al. study's (2016) confirmed that stress is perceived based on individual self-efficacy. In other words, someone's capacity to face a given life event depends on the person's perception of the event and it is circumstance-specific. The association between these two variables is negative: self-efficacy enhances people's motivation to seek more resources and effectively use these resources under stressful situations (Sumer et al., 2005; Haydon et al., 2018). Self-efficacy can take on a protective role and positively influence the organization of work and therefore help reduce stress generated by various circumstances such as work overload, students' behavioral problems, lack of control and a lack of purpose amongst others (Schwarzer and Hallum, 2008).

Studies have shown that teachers who model self-efficacy can result in students' enhanced interest in the learning process (Schunk, 1991; Caprara et al., 2006; Jones and Bouffard, 2012; Gutman and Schoon, 2013). Therefore, it can help promote students' self-esteem (Borton, 1991) motivating them to participate in class and overcome difficulties (Caprara et al., 2006; Durlak et al., 2011). Teachers' self-efficacy, however, can be influenced by a number of factors: from the perception that colleagues (Tschannen-Moran and Hoy, 2001) and students' parents/families may have on the teachers' work and capacity to obtain their educational goals (Skaalvik and Skaalvik, 2010) to a sudden change in teaching methods as has been the case during Covid-19. Teachers' self-efficacy belief may also depend on how much teachers are able to develop their transversal/soft skills which in turn makes them more efficient (Vesely et al., 2013). Because self-efficacy is related to stress perception in school settings (Schwarzer and Hallum, 2008; Chiu, 2014), the development of these skills may depend on the work context: teachers who are in supportive school settings that promote these specific skills amongst its staff and student community are able to better cope with challenging times (Hadar et al., 2020). This approach has been fundamental in protecting teachers' and students' psychological well-being (Guidetti et al., 2017; Taylor et al., 2017; Calandri et al., 2020). Based on this literature review (Luszczynska et al., 2009; Schönfeld et al., 2016), the aim of our study is to analyze the role played by self-efficacy in the relationship between the difficulties in organizing distance learning and teachers' perceived stress.

## Methods

Our study had two hypotheses:

H1: Teachers' self-efficacy mediates a positive relationship between the difficulties in organizing distance learning and perceived stress. We also considered Italy's different timing (lockdown phase) and approach/resources (distance learning implementation) when compared to other European countries, therefore:

H2: The effect of teachers' self-efficacy (mediator) in the relationship between difficulties in organizing distance learning and perceived stress is different for the two samples, Italian, and Other European Countries.

The current cross-national study examined the mediation among Difficulties in organizing Distance Learning (DDL), General Self-Efficacy (GSE), and Perceived stress (PS). Specifically, we hypothesized that: (1) DDL is positively related to PS; (2) GSE is negatively related to DDL and PS; (3) GSE mediates the relationship between DDL and PS. The same relationships were hypothesized for both samples but with different effects.

### Participants

A total of 366 teachers participated of which 86% female (age range = 23–66; mean [M] = 45.3 ± 10.37). The majority of teachers came from the North–West of Italy (IT) (55%, *N* = 200), the rest from Other European Countries (OEC) (45%, *N* = 166) of which: Germany 10%; Latvia 6%; Liechtenstein 5%; Lithuania 13%; Portugal 10%; Spain 14%; Austria 9%; Netherlands 5%; Ireland 11%; France 8%; Hungary 9%. The 89% of IT teachers and the 82% of OEC teachers were female. Teaching years of service ranged between 1 and 43, the mean for IT teachers was 14.69 (SD = 11.53) while the mean for teachers from OEC was 17.56 (SD = 10.85). At the time of administration, 6% of the total sample lived with someone who was in the medical/health field, 5% (IT) and 7% (OEC). Participants reported a 5% of positive Covid-19 cases in their family, 6% (IT) and 5% (OEC). A 43% of participants spent the lockdown period of social restriction with one or more children to care for at home, this percentage was slightly higher for IT (46%) than for OEC (40%). Most teachers judged as adequate the space to work at home (IT = 79%; OEC = 66%) and the resources to implement smart working (IT = 79%; OEC = 71%). Finally, Italian teachers (54%) were more concerned about their socio-economic situation than OEC teachers (22%).

### Procedures

Teachers compiled an online google form questionnaire which collected data on socio-demographics and on variables which are presented under the “Measures” section. The questionnaire was administered to participants in Spring 2020. The online distribution took place via email and social media through the European and local SHE (Schools for Health in Europe) networks which promote well-being at school. As a preliminary step, teachers subscribed to the informed consent. The study obtained Ethics approval from the University Bioethics Committee, Prot. n. 157942.

### Measures

#### Difficulties in Organizing Distance Learning

The difficulties in organizing distance learning (DDL) is composed of three categorical items, created *ad hoc* (evaluated with a Likert scale from 0 = not at all, to 2 = a lot), which measures difficulties to organize space and material resources, difficulties to organize timing to devote to work and work interference with family relationships. Cronbach's reliability coefficient of the present samples was 0.74.

#### Perceived Stress Scale

The Perceived Stress Scale (PSS; Cohen et al., 1983) is one of the most commonly used scales to measure the degree to which situations in one's life are appraised as stressful (Cohen et al., 1983). The 10-item scale was assessed on a 5-point Likert-type scale ranging from 0 (never) to 4 (very often). This scale was designed to be used in different settings and populations using a clear language for questions and answer options (Almadi et al., 2012). Furthermore, this scale also met our cross-cultural need since it has been tested in a number of countries (Andreou et al., 2011; Almadi et al., 2012). For the purpose of our questionnaire, we changed the wording of the introductory sentence: “during the last month” to “since the period of social distancing,” all other wording remained the same according to the original version. In the present study, Cronbach's coefficient was 0.86.

#### General Self-Efficacy Scale

The 10-item scale of General Self-Efficacy (GSE; Schwarzer and Jerusalem, 1995; Sibilia et al., 1995) was utilized to measure the participants' general sense of perceived self-efficacy. It was designed to assess optimistic self-beliefs to cope with a variety of difficult demands in life. This scale explicitly refers to personal agency, i.e., the belief that one's actions are responsible for successful outcomes. The GSE is rated on a four-point Likert scale (from 1 = not at all true to 4 = exactly true). A higher score suggests greater general self-efficacy. Cronbach's reliability coefficient of the present samples was 0.86.

#### Data Analyses

The major goal of the present study was to examine a mediation hypothesis. The study hypothesized the mediation relationship of Difficulties in organizing Distance Learning (DDL), General Self-Efficacy (GSE), Perceived Stress (PS). First, we present the result of descriptive data analyses and Pearson correlations, conducted with SPSS26, and used to examine the relationships among the variables. The mediation models were analyzed using PROCESS version 3.5 (Hayes, 2018), which was developed by (Hayes and Preacher, 2014) for SPSS. Hypotheses 1 and 2, mediation hypotheses, were tested with Hayes' Model 4. A total of 5,000 bootstraps and a confidence interval (CI) of 95% were used for estimating the effects in the PROCESS tool.

## Results

### Descriptive Analyses

Information on descriptive statistics and correlations among the variables for the total sample are reported in Table 1. Consistent with former research, all the correlations are significant among the variables, but Perceived Stress (*r* = −0.35, *p* < 0.001) and Difficulties in organizing Distance Learning (*r* = −0.42, *p* < 0.001) are negatively related to General Self-Efficacy.

**Table 1:** Descriptive statistics and correlations for study variables.

**Variable**	***n***	***M***	***SD***	**1**	**2**	**3**
(1). Perceived stress^a^	366	17.88	5.98	–		
(2). General self-efficacy^a^	366	24.29	7.10	−0.353^*^	–	
(3). Difficulties in organizing distance learning^a^	366	3.10	1.81	0.452^*^	−0.420^*^	–
(1). Perceived stress^b^	200	18.43	6.51	–		
(2). General self-efficacy^b^	200	18.99	3.59	−0.493^*^	–	
(3). Difficulties in organizing distance learning^b^	200	3.63	1.75	0.487^*^	−0.187^*^	–
(1). Perceived stress^c^	166	17.18	5.17	–		
(2). General self-efficacy^c^	166	30.90	4.27	−0.499^*^	–	
(3). Difficulties in organizing distance learning^c^	166	2.44	1.67	0.437^*^	−0.391^*^	–

* *p < 0.001*.

a *Means, standard deviation, and correlation of the study variables for total Sample*.

b *Means, standard deviation, and correlation of the study variables for Italian Sample*.

c *Means, standard deviation, and correlation of the study variables for Other European Countries' Sample*.

Table 1 reports descriptive statistics and correlation among variables for the total sample and respectively for teachers from IT and OEC. All the correlations are significant among variables. Positive relationships emerged between PS and DDL (IT *r* = 0.48, *p* < 0.001; OEC *r* = 0.44, *p* < 0.001), while a negative relationship was maintained between PS and GSE (IT *r* = −0.54, *p* < 0.001; OEC *r* = −0.45, *p* < 0.001), as well as between DDL and GSE (IT *r* = −0.20, *p* < 0.001; OEC *r* = −0.34, *p* < 0.001). We found statistically significant differences between both samples for Difficulties in organizing Distance Learning [*t*_(364)_ = 6.391; *p* = 0.001] which is higher for IT teachers, whereas General Self-Efficacy [*t*_(364)_ = −5.353; *p* = 0.001] is higher for teachers from OEC.

### Mediation Analyses

Hypothesis 1 predicted that General Self-Efficacy (GSE) mediated the relationship between Difficulties in organizing Distance Learning (DDL) and Perceived Stress (PS), as a total sample. As shown in Table 2, DDL was significantly associated with decreased GSE [B(a) = −0.747, SE = 0.109, *t* = −6.833, *p* < 0.001], and GSE was significantly associated with decreased PS [B(b) = −0.562, SE = 0.066, *t* = −8.407, *p* < 0.001], controlling for DDL (the predictor). However, the direct effect of DDL on PS, controlling for GSE (the mediator), was B(C′) = 1.136 (SE = 0.148, *t* = 7.661, *p* < 0.001) in comparison with the total effect, B(C′) = 1.556 (SE = 0.152, *t* = 10.214, *p* < 0.001). In addition, the indirect effect (ab = 0.420) was found to be significant (95% CI = [0.252, 0.616]) with a bootstrapped confidence interval that did not contain zero. Therefore, GSE partially mediated the relationship between DDL and PS, and thus, Hypothesis 1 was supported (*R* = 0.591, *R*^2^ = 0.349).

**Table 2:** Mediation analyses on total sample.

		***B***	***SE***	***t***	**LLCI**	**ULCI**	***R*^**2**^**	***F***
**Outcome variable: GSE**								
Constant		32.153	0.392	81.930^*^	31.381	32.924	0.113	46.690
Difficulties of organizing distance learning	a	−0.747	0.109	−6.831^*^	−0.962	−0.532		
**Outcome variable: PS**								
Constant		31.137	2.207	14.102^*^	26.795	35.479	0.349	97.492
Difficulties of organizing distance learning	c′	1.136	0.148	7.661^*^	0.844	1.427		
General self-efficacy	b	−0.562	0.066	−8.407^*^	−0.693	−0.430		
Indirect effect	a^*^b	0.420	0.093		0.252	0.616		

*LL, low limit; CI, confidence interval; UP, upper limit*.

*Bootstrap sample size = 5,000*.

* *p < 0.001*.

Hypothesis 2 predicted that the effect of the General Self-Efficacy (GSE) as a mediator is different for the two samples, IT and OEC. To verify hypothesis 2, the initial sample was divided into two groups (IT and OEC) and the mediation analyses was conducted separately. For both samples, the relationship between variables remained unchanged even if with different effects (Table 3). Therefore, DDL was significantly associated with decreased GSE [IT: B(a) = −0.405, SE = 0.136, *t* = −2.974, *p* = 0.0033; OEC: B(a) = −0.901, SE = 0.189, *t* = −4.765, *p* < 0.001], and GSE was significantly associated with decreased PS [IT: B(b) = −0.875, SE = 0.103, *t* = −8.435, *p* < 0.001; OEC: B(b) = −0.407, SE = 0.083, *t* = −4.896, *p* < 0.001]. The direct effects of DDL on PS were B(C′) = 1.449 for IT and B(C′) = 1.019 for OEC (IT: SE = 0.203, *t* = 7.125, *p* < 0.001; OEC: SE = 0.215, *t* = 4.737, *p* < 0.001). This is in comparison with the total effect, IT B(C′) = 1.803 and OEC B(C′) = 1.387 (IT: SE = 0.231, *t* = 7.789, *p* < 0.001; EU: SE = 0.215, *t* = 6.440, *p* < 0.001). In addition, the indirect effect (IT: ab = 0.354; OEC: ab = 0.367) was found to be significant (IT: 95% CI = [0.090, 0.667]); OEC: 95% CI = [0.148, 0.669]) with a bootstrapped confidence interval that did not contain zero. In conclusion, GSE partially mediated the relationship between DDL and PS in both samples, but the effects of single relationships are different. The indirect effect accounts for 19.6% of the total effect of DDL on PS in the IT sample, and for 26.5% of the total effect for the OEC sample. Following Clogg et al. (1995), the difference between estimated effects computed on the two samples was tested using a *Z* test. We found that B(a) is significantly stronger for OEC (*z* = 2.13, *p* = 0.03), while B(b) is significantly stronger for the Italian sample (*z* = −3.53, *p* < 0.001). Finally, neither the direct nor the indirect effect of DDL on PS were found to be significantly different between the two samples, B(C′): *z* = 1.45, *p* = 0.15; B(ab): *z* = −0.07, *p* = 0.95. For these reasons Hypothesis 2 was not fully confirmed.

**Table 3A:** Mediation analyses on Italian sample.

		***B***	***SE***	***t***	**LLCI**	**ULCI**	***R*^**2**^**	***F***
**Outcome variable: GSE**								
Constant		30.316	0.547	55.343*	29.236	31.396	0.042	8.844
Difficulties of organizing distance learning	a	−0.405	0.136	−2.974**	−0.673	−0.136		
**Outcome variable: PS**								
Constant		38.395	3.246	11.825*	31.992	44.798	0.437	76.673
Difficulties of organizing distance learning	c'	1.449	0.203	7.125*	1.047	1.850		
General self-efficacy	b	−0.875	0.103	−8.435*	−1.080	−0.670		
Indirect effect	a*b	0.354	0.148		0.090	0.667		

**Table 3B:** Mediation analyses on Other European Countries sample.

		***B***	***SE***	***t***	**LLCI**	**ULCI**	***R*^**2**^**	***F***
**Outcome variable: GSE**								
Constant		33.251	0.564	58.962^*^	32.138	34.365	0.121	22.712
Difficulties of organizing distance learning	a	−0.901	0.189	−4.765^*^	−1.275	−0.528		
**Outcome variable: PS**								
Constant		27.406	2.832	9.675^*^	21.813	32.999	0.304	35.634
Difficulties of organizing distance learning	c′	1.019	0.215	4.737^*^	0.594	1.444		
General self-efficacy	b	−0.407	0.083	−4.896^*^	−0.572	−0.243		
Indirect effect	a^*^b	0.367	0.134		0.148	0.669	

*LLCI(ULCI), Lower (Upper)Limit of Confidence Interval*.

** *p < 0.001;*

* *p < 0.005*.

## Discussion

Faced with the growing demands from the pandemic context in which teachers still had to provide schooling to their students but with no actual school where to teach and little previous experience with integrating technology in their day-to-day curricula (König et al., 2020), we expected to see an increase in teachers' perception of stress. For the purpose of this study, we took into account the organizational difficulties of distance learning (DDL) as a factor that could influence their perceived stress (PS). Given the protective role of self-efficacy (Betoret, 2006; Klassen and Chiu, 2010; Song et al., 2018), we assumed that the relationship between the difficulties of distance learning and perceived stress could be mediated by self-efficacy for both groups of teachers, Italian (IT) and those from Other European Countries (OEC). Specifically, we expected that the difficulties associated with setting up and implementing distance learning activities could have a negative effect on teachers' self-efficacy, and that this in turn could be reflected in an increased perceived stress. In fact, the results of our study show that our hypotheses are compatible with similar research in this field (Luszczynska et al., 2009): that is, there may be an indirect effect of DDL on perceived stress, a relationship mediated by teachers' self-efficacy.

We studied the relationship between DDL and PS based on the assumption that the stress which results from DDL originates not so much from the use of distance learning but rather from what it represents: an alternative to the “face-to-face” teaching, in particular regarding the organizational difficulties at a practical level (timewise and logistical). Our results confirmed H1: the mediation analyses indicate that DDL are related to an increase in PS. H2 was not fully confirmed since there were no differences between the two individual samples. One reason for this could be that all countries were suddenly faced with technological challenges at the time of school closures (Buccolo et al., 2020; König et al., 2020; Remuzzi and Remuzzi, 2020). A further factor is represented by the conciliation between the organization of teachers' work and their own family dynamics. Distance learning obliged teachers to be available in their working role throughout the day (Hebebci et al., 2020), without a real distinction between work hours/space and personal life. Teachers were involved in concurrent responsibilities, such as home schooling for their children and caring for vulnerable family members while preparing and implementing distance learning lessons for their students. These difficulties were even more relevant if we consider that the largest percentage of teachers in IT and OEC is represented by women who, in almost 40% of cases, have organized distance learning for their students while their own children, often students themselves, had lessons at the same time (Kim and Asbury, 2020).

Based on the current literature on how difficulties can negatively influence self-efficacy (Betoret, 2006; Skaalvik and Skaalvik, 2014), we also expected that the directionality of the relationship between DDL and self-efficacy remained unchanged. Therefore, it is a negative relationship: when DDL increased, self-efficacy decreased. Indeed, this was the case for both groups of teachers when considered together as well as when considered separately. The effect of DDL on self-efficacy is greater for teachers from OEC than for IT teachers. Yet, it is important to keep in mind that all European countries faced this situation with different resources and timing. In fact, by the time data was collected from the IT teachers, distance learning had already been rigorously in place for a number of weeks in the whole country whereas this was not the case for teachers from OEC who had just started distance learning and which was, in some cases, limited to certain school grades or to certain geographical areas (JRC Map., 2020). IT teachers had another disadvantage concerning the necessary skills to face stressful situations when compared to OEC: currently, the Italian school system does not foresee programs aimed at training and promoting self-efficacy at a national level (Egido Gálvez et al., 2018) resulting in a lost opportunity to be better equipped at facing stressful situations. This may explain why the effect of the negative relationship between SE and PS is different between the two groups of teachers. As a result of a low self-efficacy, there is an increase in perceived stress which is greater for IT teachers than for OEC teachers. In accordance with studies based on previous situations of crisis (see systematic review of Luszczynska et al., 2009), our first hypothesis on the mediation role of self-efficacy, even if partial, is confirmed for both sets of teachers: adding self-efficacy to the model, reduces the effect of the DDL on perceived stress. A full mediation model was not obtained, so self-efficacy is only one of the factors that affects the relationship between the DDL and PS. We could not fully confirm our second hypothesis since the direct and the indirect effects between DDL and PS were not so different between teachers from each individual sample (OEC and IT). What was different, however, concerns the negative relationship between DDL and self-efficacy, which is greater for OEC, and the negative relationship between self-efficacy and PS, which is greater for IT. In general, difficulties in distance learning therefore have a negative effect on teachers' self-efficacy. Since a low self-efficacy increases teachers' stress perception, our study can still confirm what has been highlighted by other research (Schwarzer and Hallum, 2008; Greenberg et al., 2016): that working on teachers' self-efficacy can have a positive effect on the stress levels experienced within the school system, especially if in a situation of crisis.

## Conclusion

Based on our results, we may conclude that self-efficacy can represent an important resource for teachers to manage stressful situations such as the ones generated by the Covid-19 pandemic. However, our study has some limitations: one of these concerns the recruitment of teachers for the online survey. The population that took part in our study was not so wide, and the sample may have suffered from a self-selection bias. To this we add the heterogeneous composition of one of our samples (OEC) in light of their governments' response to the pandemic. Furthermore, given the novelty of the situation, we asked *ad hoc* questions regarding distance learning hence the validity of this construct measurement was not ascertained by previous studies. A future longitudinal research could analyze the change in the relationship amongst the constructs analyzed in our study by doing a mediation and a moderation analyses especially given the numerous scientific evidence (among others, see Klassen and Kim, 2019).

Given the importance of transversal skills, it would have also been meaningful to have measured other constructs besides self-efficacy, such as self-regulation. In spite of these limitations, we can recommend that in light of the ongoing pandemic and of possible future situations of crisis, it become a priority to promote transversal skills such as self-efficacy that can reduce stress. One way of doing so is for the competent authorities including the school management to actively offer training programs which help strengthen teachers' self-efficacy.

## Data Availability Statement

The datasets generated for this study are available on request to the corresponding author.

## Ethics Statement

The study was conducted in conformity with the recommendations of the University of Turin Ethics Committee. Before starting the study, each participant concurred and signed a written informed consent in accordance with the Declaration of Helsinki. The Ethical Committee of the University of Turin approved the study (Protocol Number: N. 157942).

## Author Contributions

ER, LSL, and ADL contributed to conceptualization and investigation. ER and ADL contributed to formal analyses, MS supervised the final analyses. ER, LSL, BT, and ADL, contributed to writing — original draft. ER, LSL, MS, and ADL contributed to writing — review and editing. ER contributed to supervision. All authors contributed to the article and approved the submitted version.

## Conflict of Interest

The authors declare that the research was conducted in the absence of any commercial or financial relationships that could be construed as a potential conflict of interest.
